# Opioid Prescribing Patterns After Pediatric Adenotonsillectomy: A Bayesian Analysis of a Cross-Sectional Survey of Otolaryngologists in Canada

**DOI:** 10.1177/19160216261425531

**Published:** 2026-02-28

**Authors:** Hussein Smaily, Pierre-Hugues Carmichael, Caroline Sirois

**Affiliations:** 1 Department of Otolaryngology—Head & Neck Surgery, Université Laval, Québec City, QC, Canada; 2 Division of Pediatric Otolaryngology—Head and Neck Surgery, CHU Sainte-Justine, Montreal, QC, Canada; 3 CHU Sainte Justine Research Institute, CHU Sainte Justine, Montreal, QC, Canada; 4 Centre Hospitalier Regional du Grand Portage, CISSS du Bas-Saint-Laurent, Rivière-du-Loup, Qc, Canada; 5 Centre of Excellence on Aging of Quebec, Quebec City, QC, Canada; 6 Faculty of Pharmacy, Université Laval, Québec City, QC, Canada

**Keywords:** adenotonsillectomy, opioids, morbidity, children, pediatric otolaryngology

## Abstract

**Importance:**

Adenotonsillectomy is one of the most common pediatric surgeries, and postoperative pain management remains variable. Despite recommendations favoring non-opioid regimens, opioids continue to be prescribed, highlighting the need to better characterize current prescribing practices.

**Objective:**

To evaluate self-reported opioid prescribing practices among Canadian otolaryngologists following pediatric adenotonsillectomy and to identify surgeon-level factors associated with opioid-sparing preferences.

**Design:**

National cross-sectional survey.

**Setting:**

Members of the Canadian Society of Otolaryngology—Head & Neck Surgery.

**Participants:**

Practicing Canadian otolaryngologists involved in the perioperative care of pediatric patients undergoing adenotonsillectomy.

**Intervention or Exposures:**

Surgeon characteristics, including subspecialty training, practice setting, and surgical volume.

**Main Outcome Measures:**

The primary outcome was self-reported use of opioid-sparing postoperative analgesia following pediatric adenotonsillectomy. Secondary outcomes included opioid type prescribed and reported minimum age thresholds for opioid use.

**Results:**

Of 517 eligible otolaryngologists, 100 responded (19.3%). Overall, 73% reported preferential use of opioid-sparing analgesia. Pediatric otolaryngologists showed strong evidence of opioid-sparing practice, with a 95% posterior probability of opioid avoidance, followed by academic (87%) and high-volume surgeons (91%). Morphine was the most-commonly-prescribed opioid (82%), whereas codeine was least preferred. Reported minimum age thresholds for opioid prescribing showed a bimodal distribution.

**Conclusions:**

Most Canadian otolaryngologists surveyed reported a theoretical preference for opioid-sparing analgesia following pediatric adenotonsillectomy.

**Relevance:**

Observed practice variation, including bimodal age thresholds, highlights opportunities for opioid stewardship initiatives and quality improvement efforts. Future studies evaluating real-world prescribing behavior are needed to inform standardized postoperative pain management strategies.

## Graphical abstract

**Graphical Abstract:**
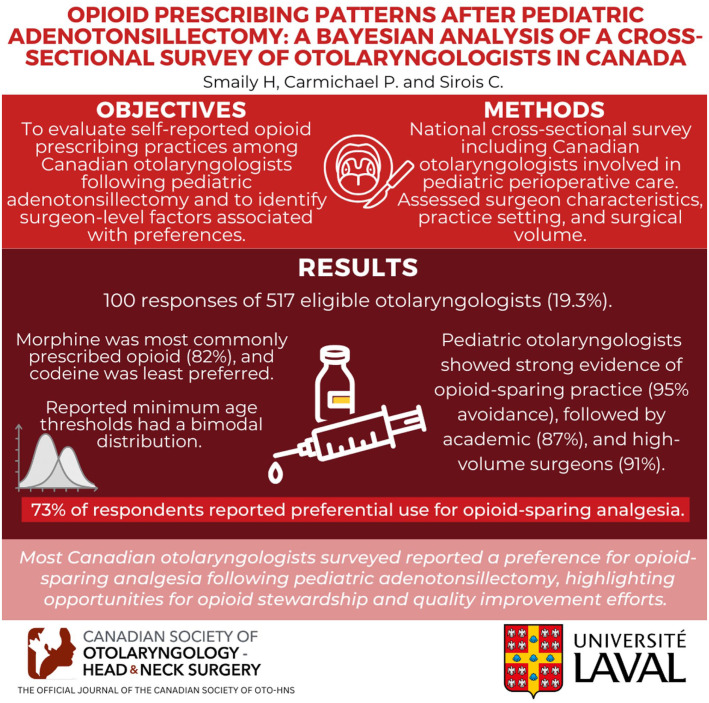

## Key Messages

- Most Canadian otolaryngologists report a theoretical preference for opioid-sparing analgesia after pediatric adenotonsillectomy.
- Substantial practice variation exists, including bimodal age thresholds for opioid prescribing.
- Findings highlight opportunities for opioid stewardship and future evaluation of real-world prescribing practices.

## Introduction

Adenotonsillectomy is one of the most-commonly-performed surgical procedures in children worldwide.^1^ Pain is a frequent associated morbidity, leading to decreased oral intake, dehydration, and need for readmission.^2^ Pain management is a core aspect of surgical care, although it was historically underappreciated. In recent years, however, the emphasis on pain assessment and treatment as key quality indicators in health care has led to a notable increase in opioid prescribing.^3^ While opioids are effective for managing acute pain, the increase in prescriptions has coincided with a growing disregard for the risks of opioid overdose, misuse,^4^ and other associated harms.^5^

In 2013, the Food and Drug Administration (FDA) issued a black box warning against the use of codeine post-adenotonsillectomy in children, after a review identified 13 deaths related to postoperative codeine use (including 8 after tonsillectomy).^6^ In 2019, the American Academy of Otolaryngology—Head and Neck Surgery’s clinical practice guidelines on adenotonsillectomy in children emphasized the use of non-opioid pain treatment regimens after adenotonsillectomy.^7^ A recent global survey of pediatric otolaryngologists reported an overall opioid prescribing rate of 28.8% among 132 respondents worldwide.^8^ Despite these recommendations, there remains considerable variability among physicians regarding optimal pain management strategies. Hence, the objectives of this survey were to (1) evaluate opioid prescribing patterns among otolaryngologists in Canada and (2) identify and evaluate potential factors associated with opioid prescription.

## Methods

### Study Design and Survey Development

We conducted a cross-sectional study using a survey developed in accordance with a validated guideline on survey design, testing, and administration.^9^ The preliminary version was pretested by 2 expert otolaryngologists to assess feasibility, clarity, ease of administration, and content validity. Revisions were made based on their feedback. Formal psychometric evaluation of the survey instrument (eg, construct validity or reliability testing) was not performed. Prior to distribution, the survey was reviewed and approved by the Electronic Communication Chair of the Canadian Society of Otolaryngology—Head and Neck Surgery (CSO-HNS). The final version comprised 14 multiple-choice and open-ended questions, requiring approximately 3 minutes to complete. It collected demographic information (eg, age of surgeon, practice setting, subspecialty interest) and data on opioid prescribing practices (Supplemental Appendix 1). We evaluated several practice-related variables as potential predictors of adopting an opioid-sparing regimen following pediatric adenotonsillectomy. These variables were selected based on clinical plausibility and included the following:

- Age of the surgeon, as a proxy for level of experience (categorized as ≤40 vs >40 years);
- Subspecialty interest (pediatric vs general otolaryngology and other subspecialties);
- Type of practice setting (academic vs community);
- Number of adenotonsillectomy performed annually (>50 vs ≤50 procedures per year); and
- Surgical technique (coblation intracapsular vs extracapsular techniques).

The study protocol was reviewed and approved by the local ethics committee (comité d’éthique de la recherche du Centre intégré de santé et de services sociaux du Bas-Saint-Laurent), under protocol number [2025-467].

### Participants

An e-mail containing a cover letter, an information sheet, and a link to access the survey on the SurveyMonkey platform was sent in June 2024 to all 517 registered otolaryngologist members of the CSO-HNS through the association’s mass mailing system. A single reminder e-mail was sent 6 weeks later to encourage participation. Participants could skip questions and select multiple answers where applicable. No personal or identifiable information was collected.

### Statistical Analysis

#### Bayesian Analytical Approach

To estimate the probability of adopting an opioid-sparing regimen and to identify factors associated with this practice, we employed a Bayesian analytical framework using the R programming language (version 4.5.0)^10^ and the rstanarm Bayesian package (version 2.32.1).^11^ A Bayesian mixed-effects model was implemented to incorporate prior knowledge from 3 previously-published studies on post-adenotonsillectomy opioid use in otolaryngology^8,12,13^ and to include a province-specific random intercept to control for geographic variability. To reflect both precision and temporal relevance, we calculated a weighted average of the reported prior probability of opioid-sparing practice, assigning greater weight to larger and more recent studies. We used 2013 as the reference year, as it marks the FDA black box warning against codeine use in children following adenotonsillectomy—a pivotal event likely to have influenced subsequent changes in clinical practice. The weight assigned to each study was computed using the following formula adapted from standard meta-analysis practice:^14^

$$w_{i}=\frac{t_{i}*\frac{n_{i}}{p_{i}\left(1-p_{i}\right)}}{\sum \;it_{i}*\frac{n_{i}}{\left(1-p_{i}\right)}}$$

where:

- *wi*: the normalized weight assigned to study *i*, reflecting both sample size and recency of the study;
- *ti*: the number of years between publication of study *i* and the FDA warning in 2013;
- *ni*: the sample size of study *i*; and
- *pi*: the proportion of opioid-sparing prescriptions reported in study *i*.

The prior probability of opioid-sparing prescribing was calculated as the weighted average of 3 prior studies, yielding an estimate of 61% with a 90% credible interval (CrI) of 10% to 97%. The prior distribution for the intercept was specified as normal, centered at logit(0.61) = 0.45, with a standard deviation (SD) of 1.18, to approximate a logistic distribution. A weakly-informative prior was used for the variability of province-specific random intercepts, specified as an exponential distribution with rate 1, given the absence of prior information on inter-provincial variability. Associations between opioid-sparing practice and clinician age, subspecialty interest, practice setting, and surgical volume were explored; all covariates were dichotomized. For these regression parameters, normal prior distributions with mean 0—reflecting no prior expectation of association—and SD 0.5 were specified. This choice was intended to yield similar prior distributions on the probability scale across modeled groups and was appropriate for all characteristics considered. Prior selection was visually assessed to ensure that the implied prior distribution for the overall probability of opioid-sparing prescribing was clinically plausible. Posterior distributions were derived using Markov Chain Monte Carlo simulation using complete respondents only, and convergence diagnostics were assessed through trace plots and the Gelman–Rubin statistic. Results are reported as posterior means with 90% Crl to avoid potential bias in estimating extreme quantiles in small samples. Figures were generated to illustrate the posterior distributions of the probability of opioid-sparing prescribing, stratified by key clinical variables. Finally, multivariable models were explored and leave-one-out computations were performed to compare the performance of these models to the univariate models using the expected log predictive density (ELPD).

## Results

**Figure 1.**
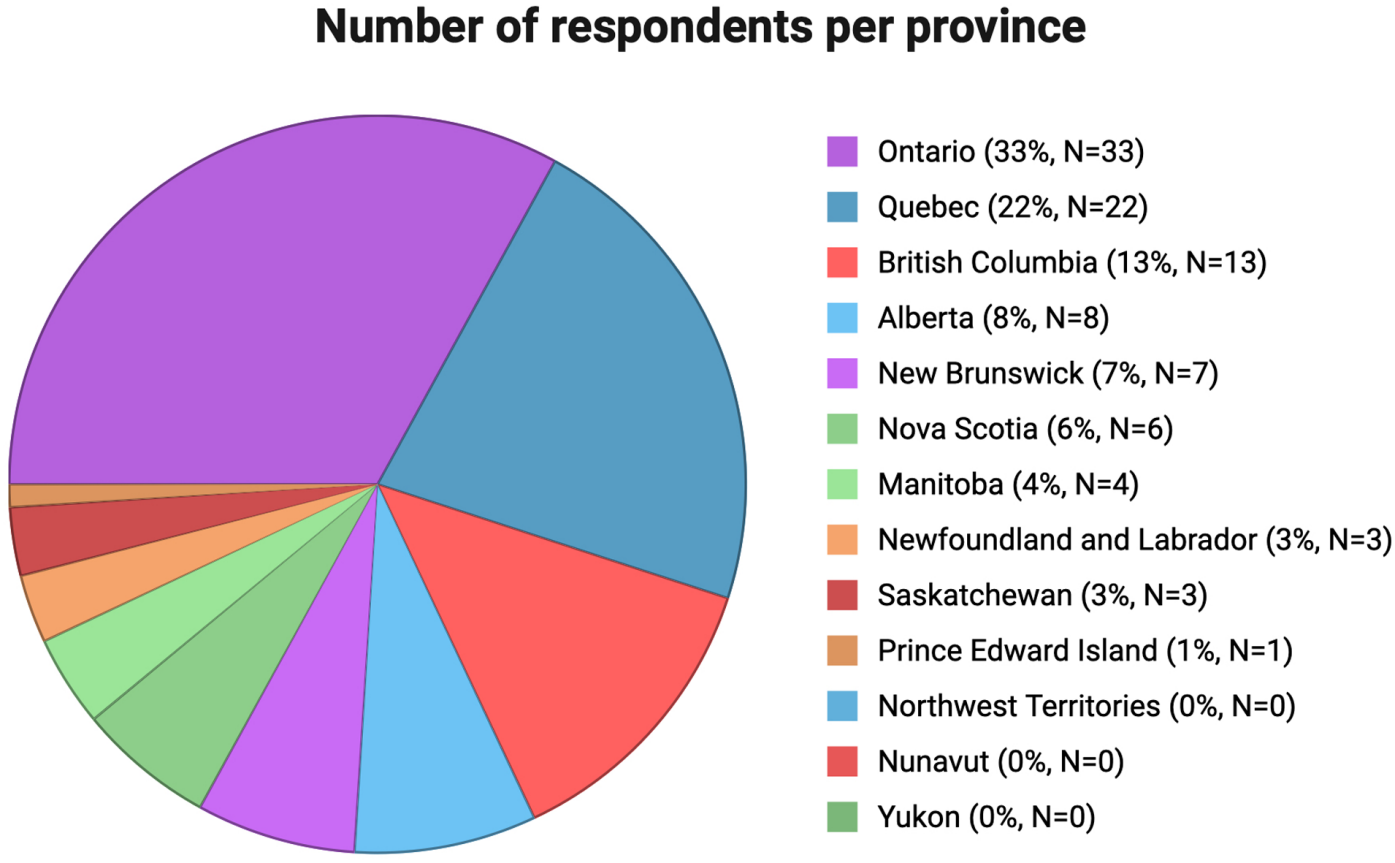
Number of respondents per province.

A total of 100 respondents completed the survey, achieving a 19.3% response rate (100/517). Respondents represented a broad geographic distribution, with the largest proportions from Ontario (33%), Quebec (22%), and British Columbia (13%) (Figure 1). Among the respondents, 40% were aged ≤40 years and 60% were aged >40 years. The majority (90%) were staff physicians, 39% work in academic-university-affiliated practices, and 24% reported a focus on pediatric otolaryngology. Full demographic characteristics are summarized in Table 1.

**Table 1.** Respondent Demographics (n = 100).

Category	n
Age	
≤40 years	40
>40 years	60
Clinical role	
Staff physicians	90
Residents	8
Retired physicians	2
Practice setting	
Academic-university affiliated	40
Community	58
Other	3
Practice interest	
General otolaryngology	68
Pediatric otolaryngology	24
Other	8
Surgical technique	
Extracapsular monopolar cautery	68
Cold steel tonsillectomy	10
Intracapsular microdebrider tonsillotomy	2
Intracapsular coblator tonsillotomy	16
Other technique	4

**Figure 2.**
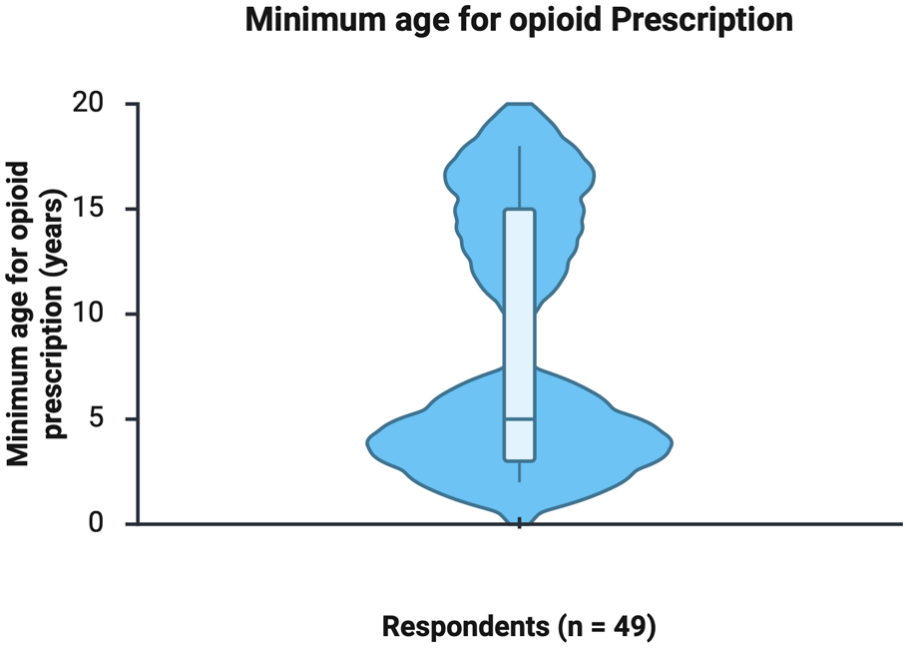
Distribution of reported minimum age for opioid prescription among 49 survey respondents. The violin plot displays the density of reported ages, with wider regions indicating more-frequently-reported values. The embedded boxplot represents the median and interquartile range. The distribution demonstrates a bimodal pattern, with 1 cluster in early childhood and a second in adolescence. The mean reported minimum age was 8.6 years (95% confidence interval: 6.9-10.3).

**Figure 3.**
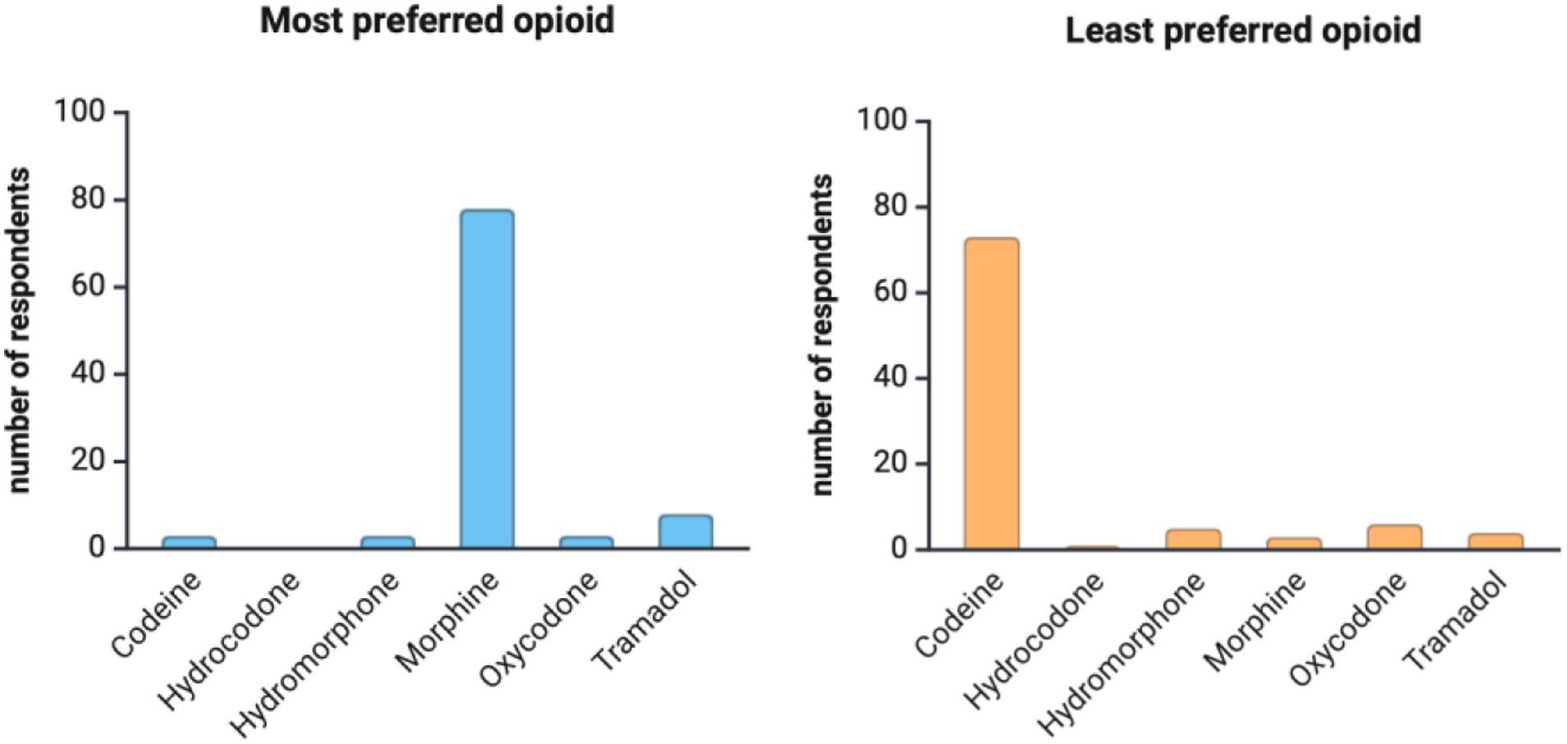
(a), (b) Most and least preferred opioid after pediatric adenotonsillectomy.

The analysis of post- adenotonsillectomy pain management practices showed that 73% of respondents reported using an opioid-sparing regimen. When asked about the minimum age for opioid prescribing, 97 physicians provided responses. Among them, 45 reported having no minimum age limit, 2 indicated they never prescribe opioids to children, and 1 reported basing the decision on a minimum weight-limit (30 kg) rather than age. For the remaining 49 respondents, the reported minimum age ranged from 2 to 18 years, with a mean of 8.6 years (SD = 5.9; Figure 2). Morphine was the most-frequently-preferred opioid medication for post-adenotonsillectomy pain management, selected by 82% of respondents. In contrast, codeine was the most frequently identified as the least preferred opioid, with 79% of respondents indicating so (Figure 3).

**Figure 4.**
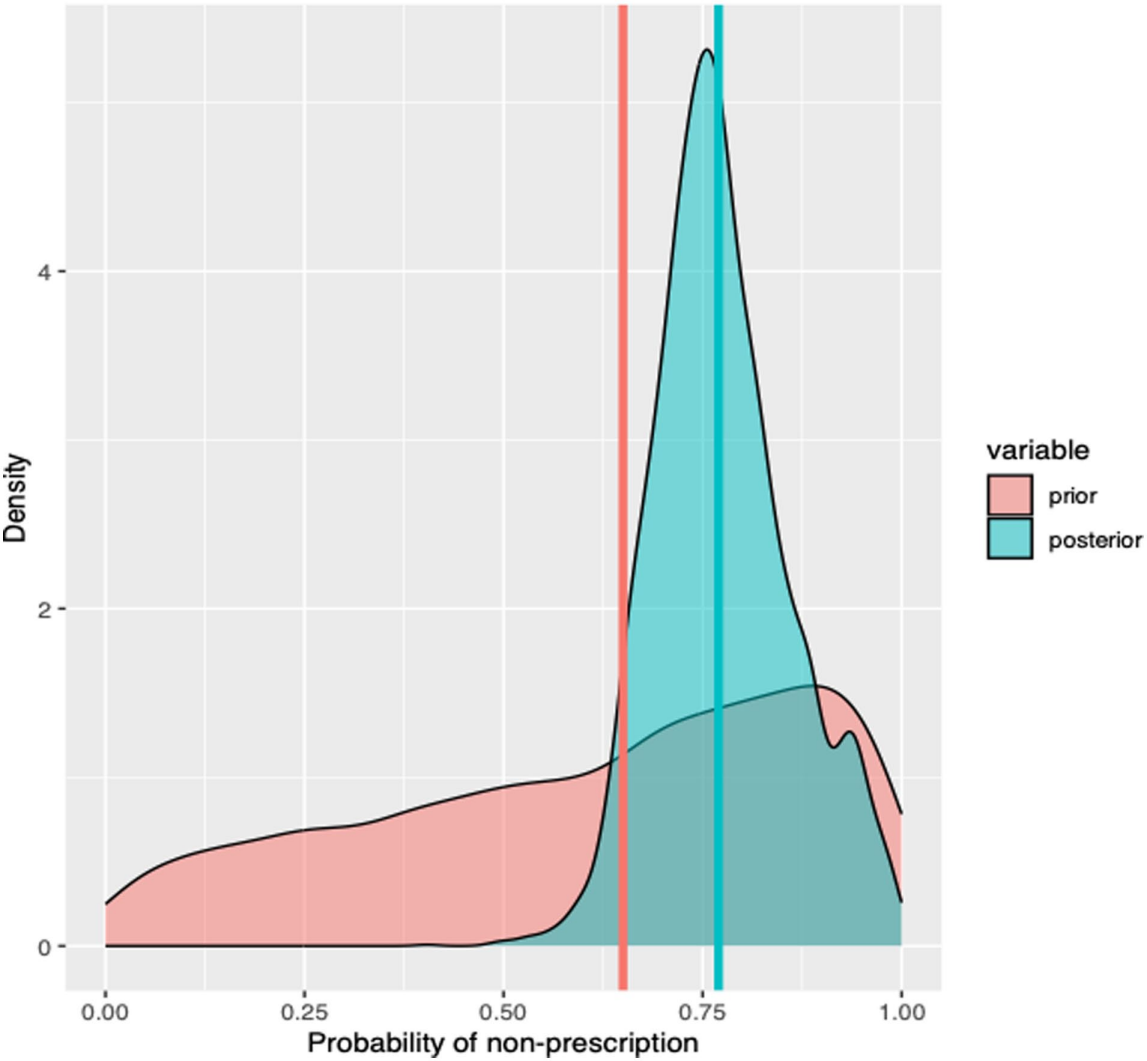
Prior probability of opioid-sparing prescription (red line) based on previous studies, and posterior probability (green line) after including the survey data (no predictors).

**Figure 5.**
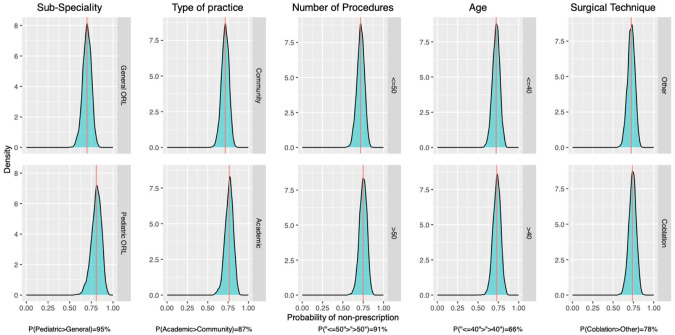
Posterior density distributions illustrating the probability of opioid-sparing prescription across clinician characteristics. Each panel compares 2 categories within a variable: (1) Subspecialty (General/other subspecialties vs Pediatric ORL), (2) Practice setting (Community vs Academic), (3) Number of adenotonsillectomy procedures per year (≤50 vs >50), (4) Age (≤40 vs >40 years), and (5) Primary surgical technique (Coblation vs Other). The probability values at the bottom of each panel indicate the probability that one group has a higher rate of opioid-sparing prescription than the other (eg, pediatric otolaryngologists were more likely to adopt an opioid-sparing regimen than those in general otolaryngology or other subspecialties with an estimated posterior probability at 95% (bottom figure: 80% (red line) CrI: [67, 90%] (green area) vs upper figure: 70% (red line) CrI: [59, 80%] (green area)).

Four respondents did not fully complete the questionnaire and were excluded from the modeling phase, leaving 96 respondents. A Bayesian mixed-effects model including a fixed intercept and province-specific random intercepts estimated the posterior probability of opioid-sparing prescribing at 77% Crl: [65%, 94%] (Figure 4). This model served as the baseline for subsequent analyses. We conducted a series of univariate Bayesian models to estimate the probability of opioid-sparing prescription across key clinician and practice characteristics (Figure 5). The posterior probability that pediatric otolaryngologists were more likely to adopt an opioid-sparing regimen than those in general otolaryngology or other subspecialties was estimated at 95% (80% CrI: [67%, 90%] vs 70% CrI: [59, 80%]). Similarly, the posterior probability that academic surgeons were more likely to adopt an opioid-sparing regimen than those in community settings was 87% (76% CrI: [68%, 84%] vs 71% CrI: [64%, 79%]). For surgeons performing more than 50 adenotonsillectomy procedures per year, the posterior probability of higher likelihood to adopt an opioid-sparing regimen compared with those performing 50 or fewer was 91% (77% CrI: [69%, 85%] vs 70% CrI: [62%, 79%]). The use of coblation techniques was associated with a slightly-higher probability of opioid-sparing prescribing than other techniques (74% vs 72%), with a posterior probability of 78%. Surgeon age showed minimal differences, with only a 66% posterior probability that surgeons aged > 40 years were more likely to adopt an opioid-sparing regimen compared with those aged ≤ 40 years (73% CrI: [65%, 80%] vs 72% CrI: [64%, 81%]). Overall, when compared to the baseline model (ELPD = −57.4), the fit was most improved when considering otolaryngology subspecialty only (ELPD = −56), and no combination of factors improved the fit (ELPD varied from −56.9 to −56.1).

## Discussion

Among Canadian otolaryngologists, an opioid-sparing approach to post-adenotonsillectomy pain management was commonly reported, with pediatric otolaryngology, academic practice, and higher surgical volume associated with a higher likelihood of adopting this strategy. However, there was no clear consensus regarding the minimum age for prescribing opioids.

The management of pain following pediatric adenotonsillectomy^15^ has evolved over time. While opioids were once considered a cornerstone for moderate-to-severe pain relief,^16^ recent trends indicate a shift toward opioid-sparing regimens. In our study, 73% of otolaryngologists in Canada reported using such regimens, consisting mainly of acetaminophen and nonsteroidal anti-inflammatory drugs. This contrasts with a 2019 survey of CSO-HNS members, in which 57.8% reported using opioid-sparing regimens after adenotonsillectomy.^13^ The higher proportion observed in our study may be attributed to the fact that the previous study included both adult and pediatric populations, whereas adenotonsillectomy tends to be more painful in adults, potentially influencing opioid use patterns. Our findings also differ from those of a 2014 survey of American Society of Pediatric Otolaryngology (ASPO) members, in which 78% of respondents reported sometimes or always prescribing opioids as a first-line analgesic.^12^ One likely explanation for this difference is the evolution of clinical practice over time. Since the ASPO survey was conducted a decade earlier, changes in clinical guidelines and institutional policies,^7,17^ increasing awareness of the opioid crisis,^18^ and the development of new surgical techniques^19^ may have contributed to a broader shift toward non-opioid alternatives. The decrease in opioid prescribing observed in our study aligns with current trends toward more cautious opioid use, especially in pediatric populations, and highlights the importance of continued efforts to optimize pain management while minimizing opioid-related risks.

Our subgroup analyses revealed noteworthy patterns: Both academic and pediatric otolaryngologists were less prone to prescribe opioids following adenotonsillectomy. This may reflect greater familiarity with the unique needs of pediatric patients and heighted awareness of opioid-related risks associated. Pediatric otolaryngologists often practice in academic or tertiary care centers, where opioid prescribing protocols tend to be more strictly applied. In contrast, general otolaryngologists, who typically treat a broader patient population (including adults), might be less familiar with the nuances of pediatric opioid safety, leading to a different approach to pain management. In another large-scale retrospective study, Goldman et al^20^ showed that academic otolaryngologists adopted the FDA’s codeine black box warning more rapidly than nonacademic otolaryngologists. Research has also shown that surgical trainees who received formal education in postoperative pain control were significantly less likely to prescribe higher opioid doses than those without it.^21^

Our study showed substantial variability among physicians regarding the minimum age for opioid prescribing. While opioids are sometimes prescribed to younger children, practices vary considerably, with some physicians opting not to prescribe them at all at younger ages. Several factors may explain this variability. First, institutional guidelines and protocols differ across hospitals and health care systems, allowing more or less flexibility in opioid prescribing (eg, setting age-based opioid restriction), leading to inconsistent practices.^22-26^ Second, awareness of risks likely play a role: Younger children are at greater risk of opioid overdose^27^ and respiratory complications,^1^ and health care providers may be more reluctant to prescribe in this age group.^28^ Third, provincial regulations and legal considerations^29^ can also shape prescribing behaviors, with stricter guidelines or monitoring programs encouraging more cautious opioid prescribing.^30^ The observed variation raises the clinically-relevant question of whether physicians who adopt higher minimum age thresholds differ systematically from those who prescribe opioids at younger ages or not at all. However, because this comparison was not a prespecified study objective and the number of respondents was limited, we did not pursue ad hoc subgroup analyses to avoid drawing unstable or potentially-misleading conclusions. Moreover, this variability is consistent with findings from other studies. A survey conducted by the American Academy of Pediatrics among pediatric surgeons showed that opioid prescribing increases with patient age after both outpatient and inpatient procedures; rates increased from 34.7 % (age < 5 years) to 54.5% (>16 years) for outpatient procedures.^31^ Similarly, a Canadian survey of pediatric surgeons showed significant variability in prescribing practice: 22% of respondents had no minimum age threshold, while 18% reported never prescribing opioids.^32^ These findings align with our own, underscoring the lack of consensus on opioid prescribing for pediatric patients. However, some important nuances are worth highlighting. Notably, a higher percentage of surgeons in the Canadian survey reported never prescribing opioids compared with our respondents (18% vs 2%). This disparity may be attributed to the nature and frequency of the surgical procedures performed: Otolaryngologists frequently carry out tonsillectomies, which are often perceived as painful procedures (moderate severity for an expected period of 10-14 days),^16^ potentially justifying more frequent opioid use. Several strategies may be implemented to standardize opioid prescribing practices after adenotonsillectomy in Canada. Benchmarking Canadian prescribing patterns against international practices may help identify evidence-based targets. The establishment of an opioid stewardship task force within the Canadian Society of Otolaryngology—Head & Neck Surgery, alongside prospective quality improvement initiatives evaluating opioid reduction and patient-centered outcomes, could support practice change. Finally, the development of Canadian, procedure-specific guidelines—particularly for pediatric adenotonsillectomy—may help reduce inter-surgeon variability.

Survey participants identified morphine as their favored opioid following adenotonsillectomy, citing several reasons: its availability in a liquid form (which facilitates administration and absorption), predictable metabolism (unlike codeine which requires liver activation), and affordability. Regulatory changes, such as warnings against codeine use in children, further solidified this preference.^33^ This finding is consistent with a previous Canadian survey of pediatric general surgeons^32^ and with the Canadian Pediatric Society position statement that oral morphine remains the drug with the strongest efficacy and safety.^34^ Moreover, most Canadian pediatric tertiary care centers favor oral morphine (easy access, lower risk of drug diversion, consistent analgesic potency).^35^ In contrast, prescribing patterns in the United States and Australia^36^ diverged after the FDA’s warning on codeine.^21^ Although codeine use declined significantly, studies showed a shift toward other opioids such as hydrocodone and oxycodone.^37^ By 2014, oxycodone had become the most-commonly-prescribed oral opioid for children in the United States.^38^ Prescriptions rose from 2 to 30% after the FDA’s warning.^39^ A survey study of American otolaryngologist also revealed that hydrocodone and codeine remained the most prescribed opioids for children undergoing otolaryngology procedures.^40^ Overall, the high rate of physician reluctance to prescribe codeine (~79%) in our survey is consistent with the numerous warnings and guidelines advising against its use in pediatric patients following adenotonsillectomy.^6,7,27,41-45^ Supporting this trend, a Canadian population–based study across 5 provinces (Alberta, Manitoba, Saskatchewan, Ontario, Quebec) between 1996 and 2021 showed that codeine safety alerts issued by Health Canada were associated with reduced exposure to codeine-containing medications among children and adolescents.^30^ These observations highlight the essential role of institutional and regulatory frameworks in shaping safe prescribing practices and emphasize the need for continuous education and adherence to updated protocols to protect pediatric patients.

This study has several strengths. Its nationwide scope provides a comprehensive overview of opioid prescribing practices across diverse clinical settings in Canada. The use of a Bayesian approach allowed for the incorporation of prior evidence and yielded informative probabilistic estimates despite a modest sample size. Furthermore, the analysis identified modifiable clinician-level and practice-level factors associated with opioid-sparing regimens, such as subspecialty interest, academic practice, and higher surgical volume. These findings may help inform targeted interventions, guideline development, and quality improvement initiatives aimed at optimizing pediatric postoperative pain management while minimizing unnecessary opioid exposure. Our study has several limitations. The response rate was 19.3%, which may limit the generalizability of the findings, as the sample may not fully represent all Canadian otolaryngologists. Selection bias is possible, as physicians who are more attuned to the opioid crisis or who favor opioid-sparing approaches may have been more likely to respond, potentially leading to an overestimation of opioid-sparing practices, including the reported 73% opioid-sparing rate. Social desirability bias may also be present due to the self-reported nature of the data; however, this is likely mitigated by the anonymity of the survey.

To preserve respondent anonymity, institutional identifiers were not collected. As a result, we were unable to determine whether multiple responses originated from the same center, and potential clustering of responses within institutions cannot be excluded, which may further limit generalizability. Although the survey instrument was reviewed by 2 experienced otolaryngologists prior to distribution, it did not undergo a formal psychometric validation process. The use of predefined questions may therefore have limited the ability to fully capture the complexity of prescribing behaviors and underlying clinical reasoning. To partially address this limitation, open-ended comments provided by respondents were included to enrich interpretation of the findings, although no formal qualitative analysis was performed. Several subgroup analyses involved relatively-small numbers of respondents. While the Bayesian hierarchical modeling approach mitigates instability through partial pooling and the use of weakly-informative priors, estimates for smaller subgroups remain less precise. Consequently, posterior probabilities for these subgroups should be interpreted with caution and viewed as exploratory rather than definitive. Finally, surgeon gender was not collected and therefore could not be examined as a potential determinant of prescribing practices, despite prior studies suggesting gender-related differences in opioid use.^46^

## Conclusion

Our findings suggest that most Canadian otolaryngologists would theoretically prefer to avoid prescribing opioids following pediatric T&A. Clinician factors such as pediatric otolaryngology subspecialty training, academic practice, and higher surgical volume are positively associated with the adoption of opioid-sparing regimens. Despite this progress, opportunities remain to improve opioid stewardship, including the standardization of pain management guidelines and exploration of alternatives for younger children to further reduce opioid use and enhance patient safety.

## Supplemental Material

sj-docx-1-ohn-10.1177_19160216261425531 – Supplemental material for Opioid Prescribing Patterns After Pediatric Adenotonsillectomy: A Bayesian Analysis of a Cross-Sectional Survey of Otolaryngologists in Canada Supplemental material, sj-docx-1-ohn-10.1177_19160216261425531 for Opioid Prescribing Patterns After Pediatric Adenotonsillectomy: A Bayesian Analysis of a Cross-Sectional Survey of Otolaryngologists in Canada by Hussein Smaily, Pierre-Hugues Carmichael and Caroline Sirois in Journal of Otolaryngology - Head & Neck Surgery
