# Supplementary material for: Opioid Prescribing Patterns After Pediatric Adenotonsillectomy: A Bayesian Analysis of a Cross-Sectional Survey of Otolaryngologists in Canada
Source: J Otolaryngol Head Neck Surg. 2026 Feb 28;55:19160216261425531. doi: 10.1177/19160216261425531 (PMC12953962; doi:10.1177/19160216261425531)
Supplement: sj-docx-1-ohn-10.1177_19160216261425531 – Supplemental material for Opioid Prescribing Patterns After Pediatric Adenotonsillectomy: A Bayesian Analysis of a Cross-Sectional Survey of Otolaryngologists in Canada [file sj-docx-1-ohn-10.1177_19160216261425531.docx]

# Appendix – Questionnaire

| Question | Question Text | Options |
| --- | --- | --- |
| 1 | In what province do you practice? | Alberta, British Columbia, Manitoba, New Brunswick, Newfoundland and Labrador, Northwest Territories, Nunavut, Nova Scotia, Ontario, Prince Edward Island, Quebec, Saskatchewan, Yukon |
| 2 | What is your category of age? | Below 30  Between 30–40  Between 40–50  Above 50 |
| 3 | What is your clinical role? | Staff physician  Resident  Fellow  Retired |
| 4 | What type of practice do you have? | University affiliated practice  Community practice  Other |
| 5 | What is your main specialty interest? | General Otolaryngology  Pediatric Otolaryngology  Other (please specify) |
| 6 | Average number of Adenotonsillectomies performed yearly? | Less than 25/year  Between 25–50/year  More than 50/year |
| 7 | Main technique used in pediatric tonsillectomies? | Extracapsular monopolar cautery  Cold steel tonsillectomy  Intracapsular microdebrider tonsillotomy  Intracapsular Coblator tonsillotomy  Other (please specify) |
| 8 | Standard home pain medication protocol? | Opioid-sparing (e.g. Acetaminophen + Ibuprofen)  Opioid-containing (e.g. Acetaminophen + Morphine) |
| 9 | Factors considered when prescribing opioids at home? | No considerations  Age of patient  Severity of Sleep Apnea  Obesity  Comorbidities  Risk of misuse/dependence  Other (please specify) |
| 10 | Do you have a minimum age limit for opioid prescription? | Yes  No  Please specify reason |
| 11 | Most preferred opioid medication (if indicated)? | Codeine, Tramadol, Oxycodone, Hydromorphone, Hydrocodone, Morphine — Please specify reason |
| 12 | Least preferred opioid medication (if indicated)? | Codeine, Tramadol, Oxycodone, Hydromorphone, Hydrocodone, Morphine — Please specify reason |
| 13 | Do you prescribe any home-steroid after Adenotonsillectomy? | Yes  No |
| 14 | Additional comments | [Open field] |
